# Supramolecular Frameworks Based on Rhenium Clusters Using the Synthons Approach

**DOI:** 10.3390/molecules26092662

**Published:** 2021-05-01

**Authors:** Nathalie Audebrand, Antoine Demont, Racha El Osta, Yuri V. Mironov, Nikolay G. Naumov, Stéphane Cordier

**Affiliations:** 1 ISCR (Institut des Sciences Chimiques de Rennes)—UMR 6226, CNRS, Univ Rennes, F-35000 Rennes, France; antoine.demont@univ-rennes1.fr (A.D.); racha.elosta@gmail.com (R.E.O.); 2 Nikolaev Institute of Inorganic Chemistry, 3 Acad. Lavrentiev pr., 630090 Novosibirsk, Russia; yuri@niic.nsc.ru (Y.V.M.); naumov@niic.nsc.ru (N.G.N.)

**Keywords:** rhenium sulfide cluster, crystal engineering, synthon, topology, supramolecular framework

## Abstract

The reaction of the K_4_[{Re_6_S^i^_8_}(OH)^a^_6_]·8H_2_O rhenium cluster salt with pyrazine (Pz) in aqueous solutions of alkaline or alkaline earth salts at 4 °C or at room temperature leads to apical ligand exchange and to the formation of five new compounds: [*trans-*{Re_6_S^i^_8_}(Pz)^a^_2_(OH)^a^_2_(H_2_O)^a^_2_] (**1**), [*cis-*{Re_6_S^i^_8_}(Pz)^a^_2_(OH)^a^_2_(H_2_O)^a^_2_] (**2**), (NO_3_)[*cis-*{Re_6_S^i^_8_}(Pz)^a^_2_(OH)^a^(H_2_O)^a^_3_](Pz)·3H_2_O (**3**), [Mg(H_2_O)_6_]_0.5_[*cis-*{Re_6_S^i^_8_}(Pz)^a^_2_(OH)^a^_3_(H_2_O)^a^]·8.5H_2_O (**4**), and K[*cis*-{Re_6_S^i^_8_}(Pz)^a^_2_(OH)^a^_3_(H_2_O)^a^]·8H_2_O (**5**). Their crystal structures are built up from *trans*- or *cis*-[{Re_6_S^i^_8_}(Pz)^a^_2_(OH)^a^_4−x_(H_2_O)^a^_x_]^x−2^ cluster units. The cohesions of the 3D supramolecular frameworks are based on stacking and H bonding, as well as on H_3_O_2−_bridges in the cases of (**1**), (**2**), (**4**), and (**5**) compounds, while (**3**) is built from stacking and H bonding only. This evidences that the nature of the synthons governing the cluster unit assembly is dependent on the hydration rate of the unit.

## 1. Introduction

Among transition metal atom clusters, hexarhenium chalcogenide cluster-based compounds have been the subject of numerous studies over several decades due to their chemical versatility and subsequent luminescence in the red-near IR window and redox or catalytic properties [1,2,3,4,5,6]. Indeed, in octahedral hexarhenium chalcogenide cluster units of general formula [{Re_6_Q^i^_8_}X^a^_6_]^n−^(Q = chalcogen and/or halogen; X = halogen, cyano or hydroxyl; *i* and *a* stand for inner and apical positions, respectively), the valence electrons are delocalized on all metal atoms of the clusters and to a lesser extent on the inner ligands offering their unique physical properties. The octahedral hexarhenium chalcogenide clusters can be used as building blocks toward various molecular assemblies, nanomaterials, and extended architectures. First, the crystal engineering based on hexa-cyano metal clusters, with formula the [{M_6_Q^i^_8_}(CN)^a^_6_]^n−^(M stands for Re and Mo), and transition or alkali metal cations has afforded extended 3D inorganic networks with a topology related to that of the Prussian blue type structure [7,8,9,10,11,12].

Secondly, the substitution of apical anionic ligands (i.e., CN^−^, OH^−^, and X^−^) by organic moieties or ligands, afterwards denoted L, has also been investigated, leading to new building blocks with general formula [{Re_6_Q^i^_8_}X^a^_6−x_(L)^a^_x_]^n−^, in order to control the self-assembling of functional clusters and then to build hybrid extended networks. The resulting literature is abundant and mainly reports on supramolecular networks involving hydrogen bonding and/or π–π stacking interactions between cluster units, and to a lesser extent coordination networks combining, for instance, the porosity of the framework with the intrinsic properties of the clusters [11]. As examples of functionalization of hexarhenium octahedral clusters with organic moieties or ligands, one can note the use of formate [13], acetate [14], phosphine [15,16,17,18], furan or thiophene dicarboxylate li-gands with Gd cation [19], and isonicotinate with lanthanide cations [20]. The last two substitutions lead to 3D frameworks through the Ln additional cations. Most of these investigations have been carried out by using amines or amino groups as functional ligands, which leads to complexes or supramolecular networks. One can note, for instance, the functionalization by azides [21,22], azolates [23,24,25,26,27], pyridine, and its derivatives [28,29,30,31,32,33,34]. When an additional cation, transition metal, or lanthanide is employed, then some extended coordination networks can be reached [35,36], as in the cases of phenantroline and Gd yielding a 2D framework [36], amines and Cu yielding a 2D framework [37], ethylenediamine and 3D transition metals yielding a 2D framework with resulting porosity [38,39], or salen salts [40].

In the course of our research dealing with extended networks built on hexarhenium chalcogenide clusters, we have focused our efforts on the cluster unit [{Re_6_Q^i^_8_}(OH)^a^_6_]^n−^ functionalized by the pyrazine ligand. Several materials have already been reported, built from H bonding, π−π interactions, and/or van der Waals contacts [28,34,41,42], sometimes with potential porosity [41]. It is still a highly challenging task to design new materials whose structures can be predicted in advance. Indeed, additional studies are required to better understand the interactions and the assembly of the building units at the nanoscale. Furthermore, the peculiarity of the [{Re_6_S^i^_8_}(OH)^a^_6_]^n−^ cluster units is their pH dependent hydration [43], which has not been explored in terms of hybrid material design. In this work, we explore the consequences of various hydration rates on the topology of the resulting hybrid materials and supramolecular networks.

Herein, we report the synthesis of supramolecular frameworks resulting from reactions between K_4_[{Re_6_S^i^_8_}(OH)^a^_6_]·8H_2_O, pyrazine (Pz) as ligand, and various nitrate and sulfate salts (Ba(NO_3_)_2_, KNO_3_, Ga(NO_3_)_3_.H_2_O, and MgSO_4_·7H_2_O) in water.

Five new compounds have been formed by slow crystallization in aqueous solution with various protonation rates: [*trans-*{Re_6_S^i^_8_}(Pz)^a^_2_(OH)^a^_2_(H_2_O)^a^_2_] (**1**), [*cis-*{Re_6_S^i^_8_}(Pz)^a^_2_(OH)^a^_2_(H_2_O)^a^_2_] (**2**), (NO_3_)[*cis-*{Re_6_S^i^_8_}(Pz)^a^_2_(OH)^a^(H_2_O)^a^_3_](Pz)·3H_2_O (**3**), [Mg(H_2_O)_6_]_0.5_[*cis-*{Re_6_S^i^_8_}(Pz)^a^_2_(OH)^a^_3_(H_2_O)^a^]·8.5H_2_O (**4**), and K[*cis*-{Re_6_S^i^_8_}(Pz)^a^_2_(OH)^a^_3_(H_2_O)^a^]·8H_2_O (**5**). The self-assembling of the hybrid cluster units is governed by supramolecular interactions increasingly dominated by H-bonding interactions as the water content rises.

## 2. Experimental

### 2.1. Synthetic Methods

All chemicals and solvents were reagent grade and were used without further purifications. The starting cluster compound K_4_[{Re_6_S^i^_8_}(OH)^a^_6_]·8H_2_O was prepared as reported previously [44].

Energy dispersive X-ray spectroscopy (EDS) was performed on single crystals at the *Centre de Microscopie Électronique à Balayage et microAnalyse* (CMEBA) at Université de Rennes 1 on a JEOL JSM 6400 microscope, equipped with an Oxford Link Isis analyzer with VARIAN SpectrAA 10 plus.

#### 2.1.1. Preparation of the Hexarhenium(III) Clusters

Preparation of a standard solution **A**: **A** was prepared by the reaction of K_4_[{Re_6_S^i^_8_}(OH)^a^_6_]·8H_2_O [44] (120 mg, 0.068 mmol) with pyrazine (C_4_H_4_N_2_), noted Pz hereafter, in a large excess (5.184 g, 64.7 mmol, Alfa Aesar) in 12 mL of distilled water. The mixture was stirred at room temperature and was placed in a PFA container (Perfluoroalkoxy) and heated at 95 °C. After six days, the resulting orange solution, of basic pH (11), was used to prepare five different hexarhenium clusters.

#### 2.1.2. Synthesis of Compounds **1** to **4**

[*trans-*{Re_6_S^i^_8_}(Pz)^a^_2_(OH)^a^_2_(H_2_O)^a^_2_] (**1**), [*cis-*{Re_6_S^i^_8_}(Pz)^a^_2_(OH)^a^_2_(H_2_O)^a^_2_] (**2**), (NO_3_)[*cis-*{Re_6_S^i^_8_}(Pz)^a^_2_(OH)^a^(H_2_O)^a^_3_](Pz)·3H_2_O (**3**), and [Mg(H_2_O)_6_]_0.5_[*cis-*{Re_6_S^i^_8_}(Pz)^a^_2_(OH)^a^_3_(H_2_O)^a^]·8.5H_2_O (**4**) were obtained from 0.5 mL of the standard solution **A** by adding different salts to yield suitable orange crystals for single crystal X-ray diffraction: Ba(NO_3_)_2_ for **1** (60.1 mg, 0.23 mmol) with resulting pH of ~9, KNO_3_ for **2** (69.69 mg, 0.69 mmol) with resulting pH of ~10, Ga(NO_3_)_3_.H_2_O for **3** (62.9 mg, 0.25 mmol) with resulting pH of ~2, and MgSO_4_.7H_2_O for **4** (56.6 mg, 0.23 mmol) with resulting pH of ~9. All solutions were left at low temperature (ca. 4 °C) for seven days. Numerous trials were necessary to optimize the synthetic routes and to obtain the compounds. In particular, the required temperature and concentration must be low to achieve crystallization of the compounds.

EDS analysis, heavy atoms ratios: (**1**): Re:S = 6:7.76; (**2**): Re:S = 6:9.6; (**3**): Re:S = 6:7.59; (**4**): Mg:Re:S = 0.89:6:7.64.

#### 2.1.3. Synthesis of Compound **5**

Orange single crystals of K[*cis*-{Re_6_S^i^_8_}(Pz)^a^_2_(OH)^a^_3_(H_2_O)^a^]·8H_2_O (**5**) were obtained after evaporation of the mixture of the **A** solution (1 mL) and KOH solution (1 mL; 2M) at room temperature. EDS analysis, heavy atoms ratio for (**5**): K:Re:S = 1.2:6:7.8.

### 2.2. Crystal Structure Determinations

Crystallographic data, details on data collections, and refinement parameters of the crystal structures are summarized in Table 1. Single-crystal X-ray diffraction data were collected at 150 K with a four-circle APEX-II Bruker-AXS diffractometer using Mo-Kα radiation (Centre de Diffractométrie X, UMR CNRS 6226, Rennes, France) and processed with the APEX 2 [45] program suite. Frame integration and data reduction were carried out with the program SAINT [46]. The program SADABS [47] was then employed for multiscan-type absorption corrections. Structures were determined by direct method using the SIR97 program [48] and then refined with full-matrix least-square methods based on F^2^ (SHELXL-2016) [49] with the aid of the WinGX platform [50]. The final refinements include anisotropic displacement parameters for the non-hydrogen atoms. The quality of the data did not allow localizing the hydrogen atoms of water molecules and hydroxyl groups. For compounds (**1**) and (**2**), the refinements of the structure frameworks have been performed by ignoring the contributions of the disordered crystallization solvent molecules. The regions containing the disordered electronic densities have been first identified by considering the Van der Waals radii of the atoms constituting the ordered framework. The contributions of these regions to the total structure factors have then been calculated *via* a discrete Fourier transformation and subtracted in order to generate new sets of *khl* reflections for each structure by means of the program SQUEEZE included in the PLATON software [51]. These new sets have been used for further least-squares refinements. Further details of the crystal-structure investigation may be obtained from the Fachinformationzentrum Karlsruhe, D-76344 Eggenstein-Leopoldshafen, Germany, on quoting the depository number(s) CSD-433379-433383 (http://www.fiz-karlsruhe.de/, 30 April 2021).

## 3. Results and Discussion

### 3.1. Crystal Structure Descriptions

The crystal structures of the five compounds have been determined from single-crystal X-ray diffraction data. Details about data collections and structure refinements are summarized in Table 1. Selected bond lengths and angles are reported in SI.

#### 3.1.1. Supramolecular Synthons Concept

The description of the crystal structures is based hereafter on the supramolecular synthon concept [52,53], in the same way as the building unit concept is widely used for the description of coordination polymers and MOFs. The term synthon has been introduced by Corey in 1967 [54] as follows: “supramolecular synthons are structural units within supermolecules which can be formed and/or assembled by known or conceivable synthetic operations involving intermolecular interactions”. The definition has been revisited later by Desiraju [52]: “supramolecular synthons are spatial arrangements of intermolecular interactions and play the same focusing role in supramolecular synthesis that conventional synthons do in molecular synthesis”. One can note that Pénicaud et al. described the crystal structures of a series of metallic organic–inorganic solvates based on Re_6_Se_5_Cl_9_ cluster units, BEDT-TTF-conducting organic cation radical slabs, and solvent as guest molecules through C-H…O hydrogen bonds that anchor guest molecules (THF, dioxane) to the organic slab [55]. The authors demonstrated the key role of these H bonds that are central to the interplay of structural ordering and conductivity.

In the crystal structures of the five compounds reported in the present study, various supramolecular synthons as well as cluster units based on the {Re_6_S^i^_8_} core are involved. One can list H bonds, strong and weak ones, interactions, and two kinds of bridges, based on NO_3_^−^ or H_3_O_2_^−^ anions. The latter anion that is named bi-hydroxide anion, hydroxide hydrate, or hydrogen oxide bridging ligand deserves to be further described. The H_3_O_2_^−^ bridges were first mentioned, to the best of our knowledge, by Abu-Dari et al. in 1979 [56]. This synthon involves H bonds classified by Desiraju as strong bonds [57]. H_3_O_2_^−^ anions can be seen as bridging ligands in which the O-O bond length is very short and varies between 2.44 and 2.56 A [58,59,60,61]. The H_3_O_2_^−^ ligand is formed from one water ligand that may hydrolyze to a hydroxo ligand, leading to a strong H bond between the hydroxo ligand of one ion and an aquo ligand of another ion [60,61]. The first example of a µ-H_3_O_2_^−^ bridging ligand in a MOF material has been reported by Monge et al. [62]. Several examples of cluster-based materials are built from supramolecular interactions and involve strong extended hydrogen bonding like H_3_O_2_^−^ bridges. One can cite octahedral rhenium cluster complexes, which are related to the present work, as [{Re_6_Q^i^_8_}(H_2_O)^a^_n_(OH)^a^_6−n_]^n^^−4^ (Q: S, Se) [43], *trans*-[{Re_6_S^i^_8_}(CN)^a^_4_(OH)^a^_2-n_(H_2_O)^a^_n_]^n−4^ [63], or [{Re_6_S^i^_8_}(OH)^a^_2_(bpy)^a^_4_]·bpy·4H_2_O [34].

#### 3.1.2. A Common Cluster-Based Building Block

**Figure 1:**
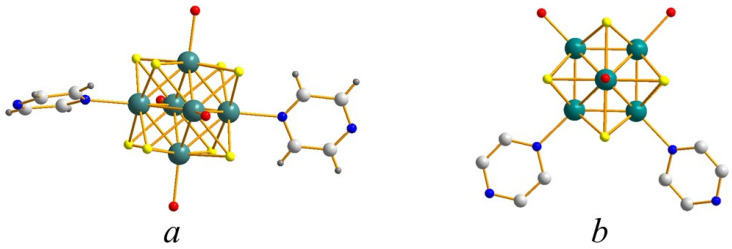
View of the cluster unit [Re_6_S^i^_8_(Pz)^a^_2_(OH)^a^_4−x_(H_2_O)^a^_x_]^x−2^ in (**a**) *trans* and (**b**) *cis* configurations. Red atoms stand for either hydroxyl groups or water molecules.

The crystal structures described herein are based on [{Re_6_S^i^_8_}(Pz)^a^_2_(OH)^a^_4−x_(H_2_O)^a^_x_]^x−2^ building blocks that result from the grafting of two pyrazine ligands on the Re_6_ octahedral cluster core of {[Re_6_S^i^_8_}(OH)^a^_6_]^4−^ cluster units by the substitution of two hydroxyl groups. These units can be depicted as octahedral Re_6_ clusters face-capped by eight inner sulfur ligands (μ_3_-S). The position of the organic ligands leads to cluster units with either a *trans* (compound **1**) or *cis* (compounds **2**–**5**) configuration (Figure 1).

When comparing the interatomic distances in the five crystal structures, one can notice that the bond lengths within the cores are homogeneous and have similar values. The Re–Re and Re–S bond lengths within the cluster core vary from 2.571(1) to 2.604(1) and 2.401(3) to 2.453(6) Å, respectively. Additionally, the Re–N bond lengths range from 2.12(2) to 2.22(2) Å. Finally, within the cluster units, the Re–O bond lengths range from 2.058(9) to 2.15(1) Å. It is not possible to distinguish the rhenium atoms coordinated with OH^−^ from those coordinated with water molecules, since the H atoms have not been localized. These distances agree well with those found, for instance, in neutral *trans*-[{Re_6_Q^i^_8_}(TBP)^a^_4_(OH)^a^_2_] (TBP = *p*-*tert*-butylpyridine) [29] and [{Re_6_S^i^_8_}(OH)^a^_2_(bpy)^a^_4_]·bpy·4H_2_O and [{Re_6_S^i^_8_}(OH)^a^_2_(apy)^a^_4_]·2apy·2H_2_O [34].

#### 3.1.3. Crystal Structures of Compounds **1** to **5**

Depending on the syntheses conditions and given the partial substitution of pyrazine for hydroxyl groups, the protonation can be observed on the remaining hydroxyl groups. Hence, the formula of the building blocks is [{Re_6_S^i^_8_}(Pz)^a^_2_(OH)^a^_4−x_(H_2_O)^a^_x_]^x−2^, with x a pH dependent variable. Such an acido-basic behavior is reminiscent of other hydroxyl containing rhenium clusters and was for example characterized in details for [{Re_6_S^i^_8_}(OH)_6−x_(H_2_O)_x_]^x−4^ species (also written [{Re_6_S^i^_8_}(OH)_6−x_(H_2_O)_x_]^(4−x)−^) [43,64]. The latter species are highly related to those of the present study, since they can be seen as the non-substituted equivalent of the [{Re_6_S^i^_8_}(Pz)^a^_2_(OH)^a^_4−x_(H_2_O)^a^_x_]^x−2^ building blocks. One can notice that selenium analogous [{Re_6_Se^i^_8_}(OH)^a^_2_(H_2_O)^a^_4_] [65] and [{Re_6_S^i^_8_}(OH)^a^_2_(H_2_O)^a^_4_] [64] species have also been reported. Compounds (**1**) and (**2**) are built upon [{Re_6_S^i^_8_}(Pz)^a^_2_(OH)^a^_4−x_(H_2_O)^a^_x_]^x−2^ cluster units with x = 2 and therefore consist of the self-assembling of neutral [{Re_6_S^i^_8_}(Pz)^a^_2_(OH)^a^_2_(H_2_O)^a^_2_] motifs. The resulting architectures are primarily governed by H bonding. In contrast, the architectures of compounds (**3**), (**4**), and (**5**) rely on several types of interactions, which include stacking, H bonding, and Coulomb forces.

##### Compound (**1**)

**Figure 2:**
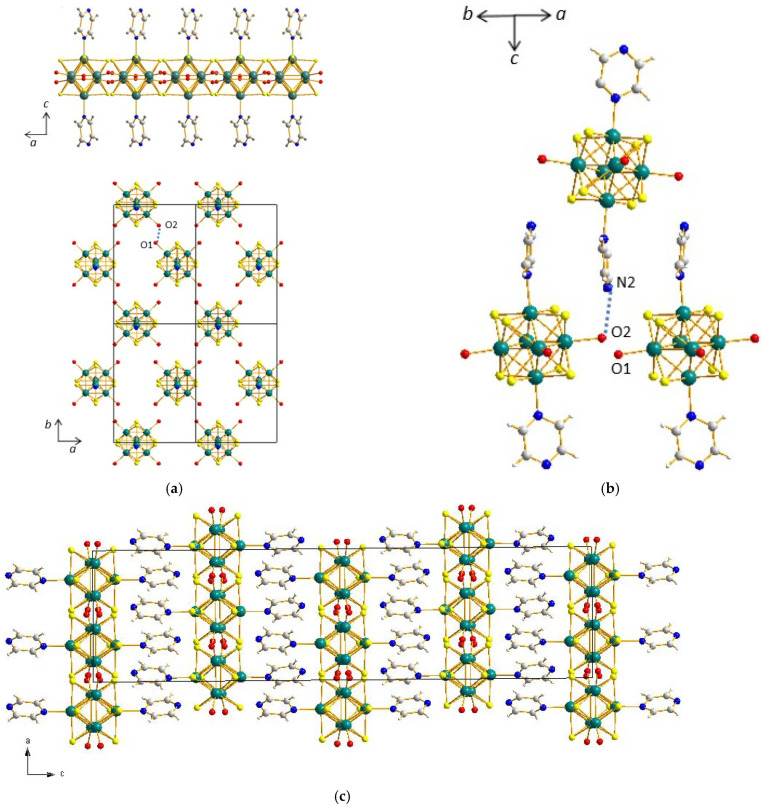
(**a**) Projections along the *b* and *c* axes of the structure of (**1**) depicting a two-dimensional network based on H_3_O_2_^−^ bridges. (**b**) View of the structure of (**1**) showing hydrogen bonds along the *c* axis. (**c**) Projections along the *b* axis of the supramolecular structure of (**1**).

The structure of (**1**) relies on three different synthons: [{Re_6_S^i^_8_}(Pz)^a^_2_(OH)^a^_4−x_(H_2_O)^a^_x_]^x−2^ building block, H_3_O_2_^−^ bridges, and H bonds. Due to the trans configuration of the pyrazine molecules grafted on their Re_6_ core (Figure 1a), the *trans-*{Re_6_S^i^_8_}(Pz)^a^_2_(OH)^a^_2_(H_2_O)^a^_2_ cluster units (x = 2), which are the first synthon, show a pseudo-plane containing four equatorial oxygen atoms. From each oxygen atom of this pseudo-plane, a strong hydrogen bond (O1–O2 bond length of 2.47(1) Å) connects the cluster to another one. Each cluster is therefore connected to four other clusters with four symmetrically equivalent hydrogen bonds, which allows the formation of an infinite two-dimensional network parallel to the (001) plane and based on H_3_O_2_^−^ bridges as the second type of synthon (Figure 2a), as suggested by d_O-O_ < 2.50 Å.

The third synthon (Figure 2b) for these neutral trans [{Re_6_S^i^_8_}(Pz)^a^_2_(OH)^a^_2_(H_2_O)^a^_2_] cluster units is made of a different hydrogen bond along the *c* axis involving each terminal nitrogen atoms of both aromatic rings of one cluster unit and an equatorial oxygen atom from a different cluster unit, with d_N2-O2_ = 2.88(2) Å. Owing to the infinite and one-dimensional connectivity of the additional H bond in the second synthon, the supramolecular sheets formed with the first synthon are stacked along the *c* axis, resulting in the supramolecular assembly of (**1**) (Figure 2c). Additionally, there are no interactions between neighbored pyrazine groups, since the centroid–centroid distance is too high (4.71 Å).

##### Compound (**2**)

**Figure 3:**
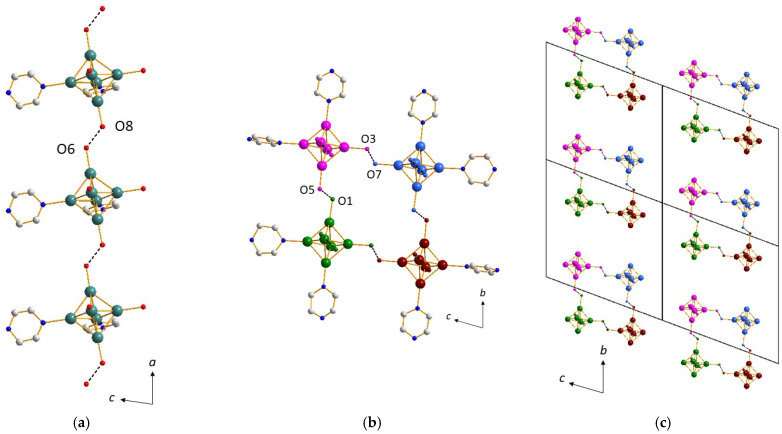
(**a**) Infinite chain through strong H-bonding along the *a* axis in the structure of (**2**). (**b**) Connection of adjacent chains through H_3_O_2_^−^ bridges in the structure of (**2**) evidencing clusters tetramers. (**c**) Infinite tunnels running along the *a* axis in the structure of (**2**).

**Figure 4:**
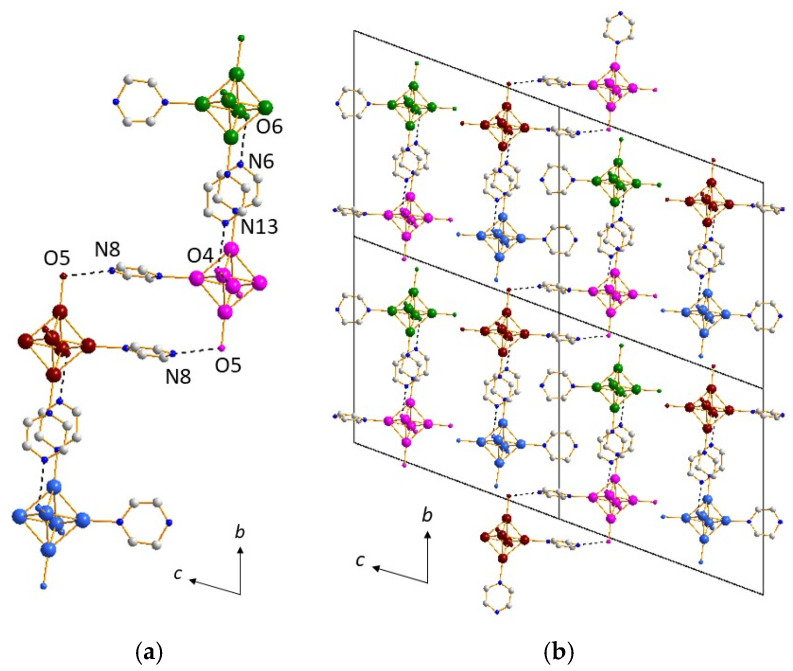
(**a**) Connection of the tunnels through H bonds involving the pyrazine cycles in the structure of (**2**). (**b**) Projection along the *a* axis of the supramolecular framework of (**2**).

The structure of *cis*-[{Re_6_S^i^_8_}(Pz)^a^_2_(OH)^a^_2_(H_2_O)^a^_2_] clusters (x = 2) (**2**) is based on four different synthons, involving H bonding. The first synthon is the neutral *cis-*{Re_6_S^i^_8_}(Pz)^a^_2_(OH)^a^_2_(H_2_O)^a^_2_ cluster unit (x = 2). The second synthon gives rise to two infinite chains, along the *a* axis, of clusters connected by hydrogen bonds with d_O2-O4_ = 2.43(2) and d_O6-O8_ = 2.46(2) Å, which are characteristic of H_3_O_2_^−^ bridges (Figure 3a). A third synthon allows for the connections between these adjacent chains, through H bonding again, with d_O1-O5_ = 2.48(1) and d_O3-O7_ = 2.56(2) Å, revealing the presence of H_3_O_2_^−^ bridges (Figure 3b) and forming clusters tetramers. The chains are then linked four by four, and the two described synthons yield to the formation of infinite tunnels running along the *a* axis (Figure 3c). Tunnels are connected together by a fourth synthon (Figure 4a), which involves the pyrazine cycles in H bonding with one oxygen atom of the cluster and with a d_N-O_ of c.a. 2.85 Å (d_N6-O6_ = 2.85(2), d_N8-O5_ = 2.84(2), and d_N13-O4_ = 2.85(2) Å). It is worth noting that although a superimposition of the aromatic cycles is observed along the *a* and *c* axis, π–π stacking is not governing the formation of this third synthon, with d_c-c_ c.a. 4.75 Å. Therefore, the resulting framework of compound (**2**) (Figure 4b) is mainly resulting from H-bonding interactions.

In contrast to the aforementioned compounds (**1**) and (**2**) based on neutral [{Re_6_S^i^_8_}(Pz)^a^_2_(OH)^a^_2_(H_2_O)^a^_2_]^0^ cluster units, compounds (**3**), (**4**), and (**5**) contain charged cluster units and have structures that rely on several types of interactions, which include H bonding as compounds (**1**) and (**2**), but also π–π stacking and Coulomb forces for (**3**), (**4**), and (**5**). The [{Re_6_S^i^_8_}(Pz)^a^_2_(OH)^a^_4−x_(H_2_O)^a^_x_]^x−2^ cluster units are positively charged in (**3**) (x = 3) and negatively charged in (**4**) and (**5**) (x = 1), leading to the respective formulae [{Re_6_S^i^_8_}(Pz)^a^_2_(OH)^a^(H_2_O)^a^_3_]^+^ and [{Re_6_S^i^_8_}(Pz)^a^_2_(OH)^a^_3_(H_2_O)^a^]^−^. The counter ions involved in (**3**), (**4**), and (**5**) are, respectively, NO_3_^−^, Mg^2+^, and K^+^ ions.

##### Compound (**3**)

**Figure 5:**
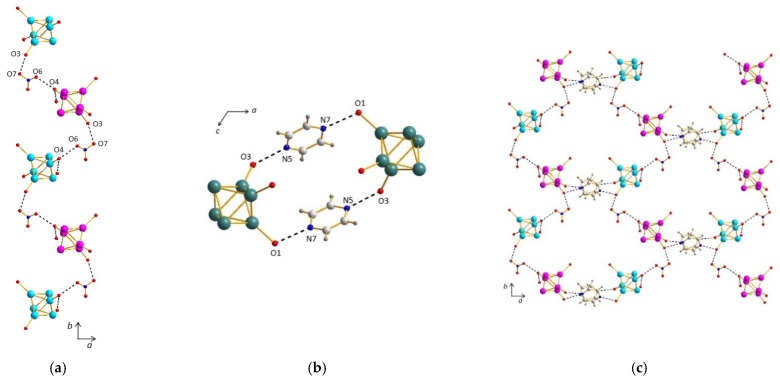
(**a**) Infinite chains of clusters bridged by nitrate groups along the *b* axis in the structure of (**3**). (**b**) Strong H bonding involving free pyrazine molecules leading to interchain connectivity and formation of dimers in the structure of (**3**). (**c**) Infinite sheet in the (*ab*) plane resulting from H bonds involving pyrazine molecules and nitrate bridges in the structure of (**3**).

**Figure 6:**
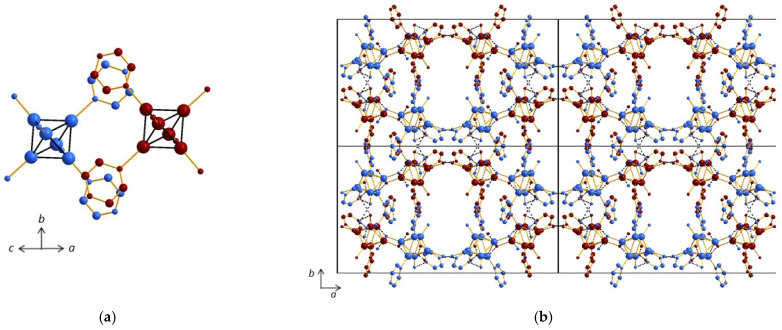
(**a**) π–π stacking involving coordinated generating cluster dimers in the structure of (**3**). (**b**) Stacking of the sheets along the *c* axis in the structure of (**3**). Crystallization water molecules are not depicted for clarity.

A complex architecture was observed in the case of compound (NO_3_)[*cis-*{Re_6_S^i^_8_}(Pz)^a^_2_(OH)^a^(H_2_O)^a^_3_](Pz)·3H_2_O (**3**), with nitrate groups and an excess of pyrazine incorporated in the structural framework. Four synthons based on three different types of interactions govern the architecture. The first one is the cluster unit [*cis-*{Re_6_S^i^_8_}(Pz)^a^_2_(OH)^a^(H_2_O)^a^_3_]. The second one shows clusters connected by nitrate bridges, through H bonds, with d_O3-O7_ = 2.75(2) Å and d_O6-O4_ = 2.66(3) Å from one free nitrate group to two cluster units, which results in the formation of an infinite zigzag chain running along the *b* axis (Figure 5a). The excess of pyrazine is involved in a third synthon that ensures chain connectivity owing to strong H bonding between one pyrazine group with two cluster units, with d_O3-N5_ = 2.56(3) Å and d_O1-N7_ = 2.59(4) Å (Figure 5b). Both nitrogen atoms of the crystallization pyrazine are involved in the supramolecular bonding, and the synthon therefore consists in a dimer containing two clusters and two crystallization pyrazine molecules (Figure 5b, Figure S1). The combination of these dimers with the aforementioned nitrate bridges allows for the formation of an infinite sheet in the (*ab*) plane (Figure 5c). The fourth synthon (Figure 6a) involves π–π stacking with d_Centroid–Centroid_ < 3.6364(2) Å from pyrazine groups and consists of dimers of clusters that enable connections between the infinite sheets (Figure 6b), which are then stacked along the *c* axis.

Then, the supramolecular framework of compound (**3**) results from the combination of four synthons (Figure 6b and Figures S2 and S3). Furthermore, the structure contains three crystallization water molecules and implies as a consequence additional H bonds. Whereas OW2 is only connected to OW1 molecule through a very weak H bond (d_OW1-OW2_ = 3.17(6) Å), OW1 is linked to the O4 atom, which is already involved in the second synthon, implying the nitrate groups (d_OW1-O4_ = 2.56(2) Å), and OW3 is linked to O7 and O1 atoms, which are already involved in the second and third synthons, implying nitrate groups and crystallization pyrazine molecules, respectively (d_OW3-O7_ = 3.00(6) Å, d_OW3-O1_ = 2.46(5) Å). Therefore, even if these three water molecules are not essential for the construction of the 3D network, they reinforce its stability.

##### Compound (**4**)

**Figure 7:**
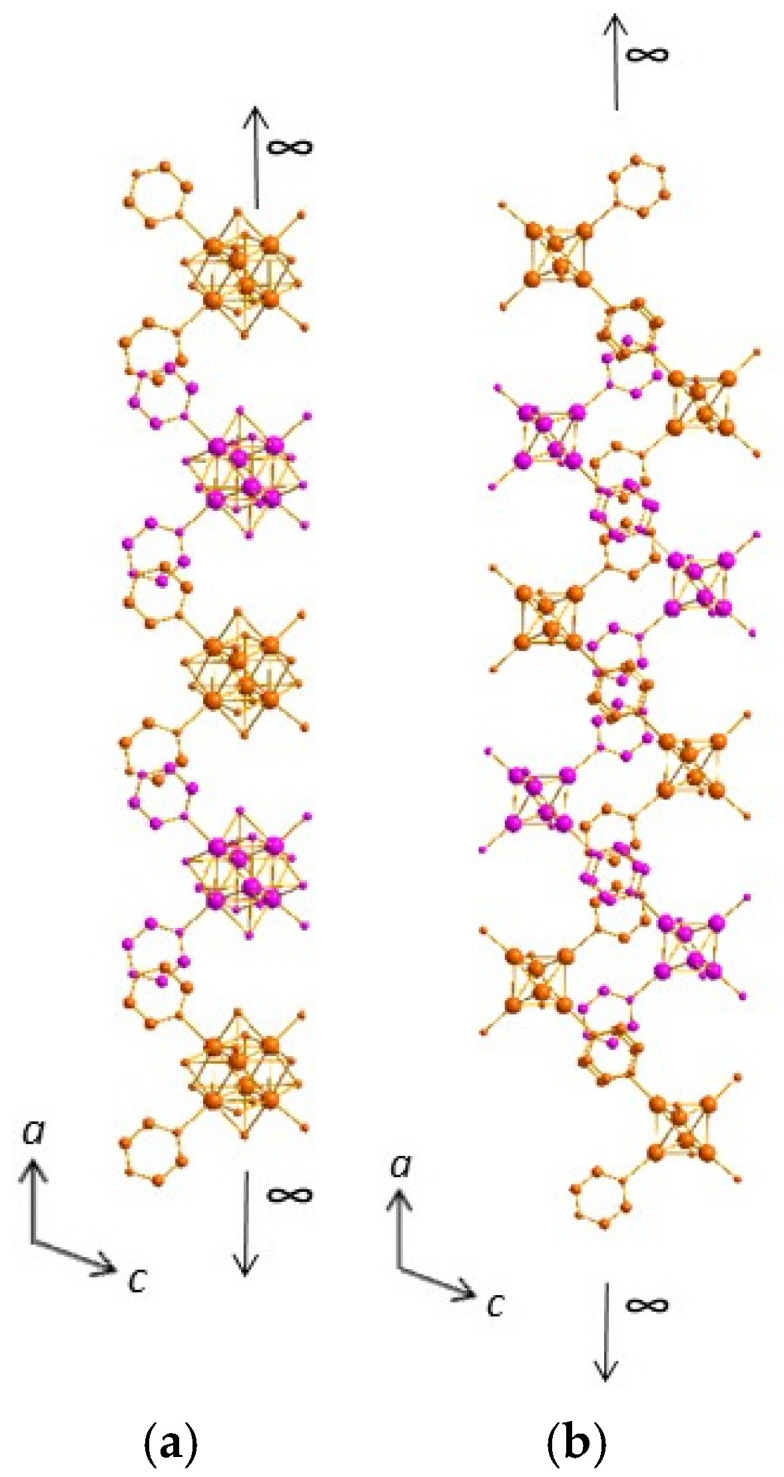
(**a**) Infinite chain of cluster units linked by π–π stacking involving adjacent coordinated pyrazine molecules along the *a* axis in the structure of (**4**). Clusters on the top and clusters on the bottom of the figures are depicted in two different colors (orange and pink). (**b**) View of two pillared adjacent infinite chains in the structure of (**4**).

**Figure 8:**
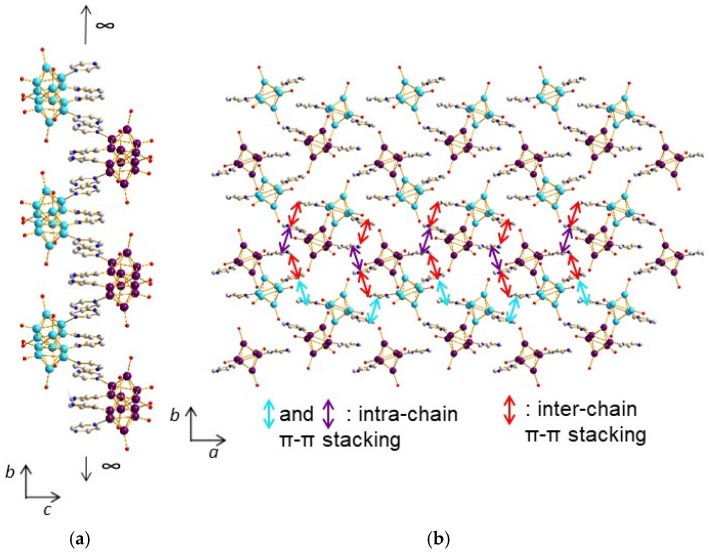
(**a**) Stacking of the chains along the *b* axis through π–π interactions involving adjacent coordinated pyrazine molecules in the structure of (**4**). (**b**) Infinite two-dimensional layers resulting from intra- and inter-chain π–π stacking of the aromatic cycles in the structure of (**4**).

In compound (**4**) [Mg(H_2_O)_6_]_0.5_[*cis*-Re_6_S^i^_8_(Pz)^a^_2_(OH)^a^_3_(H_2_O)^a^]·8.5H_2_O, the aromatic cycles of the pyrazine groups linked to the cluster units are dramatically involved in the cluster units’ self-assembly, and then the supramolecular assembly is mainly built from π–π stacking interactions. The first synthon is the cluster unit [*cis*-Re_6_S^i^_8_(Pz)^a^_2_(OH)^a^_3_(H_2_O)^a^]. On one hand, two types of synthons based on π–π stackings govern the formation of supramolecular two-dimensional ribbons. The first one allows both aromatic rings of one cluster unit to connect with two different adjacent cluster units (the d_centroid-centroid_ are 3.65 and 3.79 Å between pyrazine groups), leading to the formation of an infinite chain along the *a* axis (Figure 7a and Figure S4), as the interaction extends from one cluster to another one. Within this chain, half of the clusters are located slightly above and others slightly below the central axis of the chain (Figure S4). Another synthon involves both aromatic rings of one cluster unit into a connection with only one another cluster unit belonging to another chains (d_centroid-centroid_ are 3.65 and 3.75 Å) (Figure 7b and Figure S5), which results in cluster dimers (Figure S5) and then the stacking of the chains along the *b* axis (Figure 8a). The combination of both synthons, with intra- and inter-chain stacking, results in the formation of the infinite two-dimensional layers based on the π–π stacking of the aromatic cycles (Figure 8b).

**Figure 9:**
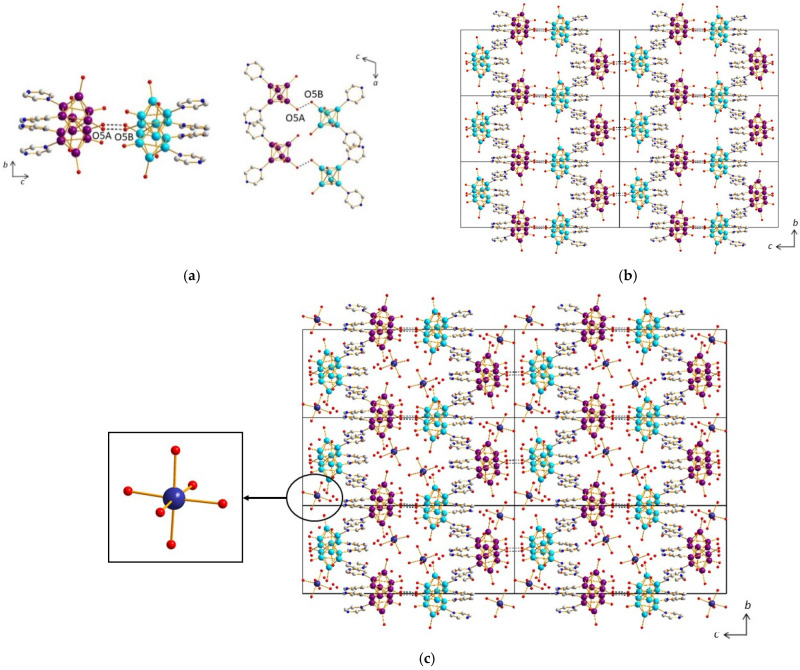
(**a**) H_3_O_2_^−^ bridges in the structure of (**4**) promoting the formation of cluster dimmers. (**b**) Stacking of the layers yielding to the three-dimensional supramolecular framework of (**4**). (**c**) View of the structure of (**4**) along the *a* axis showing insertion of Mg(H_2_O)_6_ octahedra into the voids of the supramolecular framework.

On another hand, a strong H bond (d_O5A-O5B_ = 2.50(1) Å) is observed, with distance and angles characteristic of H_3_O_2_^−^ bridges, which promotes the formation of cluster dimmers (Figure 9a). This synthon ensures the layers connectivity along the *c* axis and allows for the stacking, yielding to a three-dimensional supramolecular framework constitutive of the architecture of compound (**4**) (Figure 9b, Figure S6).

This supramolecular negatively charged framework generates voids in which Mg^2+^ cations are incorporated in the form of [Mg(H_2_O)_6_]^2+^ octahedral complexes (Figure 9c), along with crystallization water molecules. The crystallographic unit cell contains 4 Mg^2+^ per 8 [{Re_6_S^i^_8_}(OH)^a^_3_(H_2_O)^a^Pz^a^_2_]− cluster units, i.e., a ratio of ½, as well as 68 crystallization water molecules, all atoms sitting on 4*e* Wickoff position. There are numerous H bonds, sometimes relatively strong (d_O-O_ ~ 2.7 Å), between on one hand, the water molecules coordinating the magnesium cations with crystallization water molecules and oxygen atoms coordinated to rhenium (O3A, O3B and O4A), and, on the other hand, between crystallization water molecules themselves. These H bonds reinforce the supramolecular network. One can note that in the structure of (**4**), magnesium cations are not coordinated to the cluster units but on the contrary to metal-based hexahydroxo rhenium cluster, where metal stands for alkaline earth metals including Mg, reported in the literature [66]. In the structures of molecular hexanucluear chalcohalide rhenium clusters, aqua-calcium or aqua-magnesium ions are connected to each other through hydrogen bonds, forming cavities encapsulating the rhenium cluster anions [67].

##### Compound (**5**)

**Figure 10:**
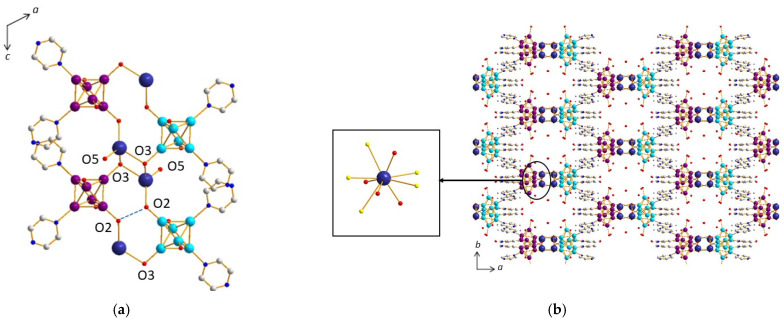
(**a**) Strong H bonds providing the layers’ connectivity in the structure of (**5**). (**b**) View of the structure of (**5**) along the *c* axis showing insertion of KO_4_S_5_ polyhedra into the voids of the supramolecular framework.

The structure of compound (**5**), namely K[*cis*-{Re_6_S^i^_8_}(Pz)^a^_2_(OH)^a^_3_(H_2_O)^a^]·8H_2_O, is highly related to that of (**4**) (Figure 10b). It is based on similar supramolecular layers whose cohesion is governed by two kinds of π–π stacking interactions. The first synthon is the same as in compound (**4**), i.e., the cluster unit [*cis*-Re_6_S^i^_8_(Pz)^a^_2_(OH)^a^_3_(H_2_O)^a^]. At first, chains are built from a second synthon (d_centroid-centroid_ of 3.68 Å between pyrazine groups of adjacent cluster units) along the *c* axis (equivalent to *a* axis in compound (**4**)), which are then pillared along the *b* axis thanks to a third synthon and to the formation of cluster dimers (d_centroid-centroid_ = 3.68 Å). One should note that only one d_centroid–centroid_ distance is observed for each synthon compared to (**4**), where two distances are in each case involved. The reason is simply the higher symmetry of (**5**) (*C*2/*c*) when compared to (**4**) (*P*2_1_/*c*). Comparing the structures of (**4**) and (**5**) compounds, the main difference arises from the layers’ connectivity involving, in the case of (**5**), not only a strong H bond (d_O2-O2_ = 2.81(1) Å) (Figure 10a) but also K^+^ cations (Figure 10a,b), which are incorporated in between the layers in the form of a KO_4_S_5_ polyhedron (Figure 10b). The crystallographic unit cell contains 8 K^+^ per eight [{Re_6_S^i^_8_}(OH)^a^_3_(H_2_O)^a^Pz^a^_2_]− cluster units, i.e., a ratio of 1, ensuring the electro-neutrality of the crystal structure. Among the four O atoms surrounding the K^+^ cation, three are linked to Re atoms, leading in that case to a coordination network. One of them belongs to a water molecule. The unit cell contains additionally seven crystallization water molecules per cluster unit. The eight water molecules are, as in the case of compound (**4**), involved in numerous H bonds, sometimes relatively strong (d_O-O_ ~ 2.7 Å), with oxygen atoms coordinated to rhenium (O1, O2, O3, O4), on the one hand, and between the water of crystallization molecules with each other, on the other hand. These H bonds reinforce the supramolecular network. Although water molecules are also present in the voids, coordinating clusters through weak H bonds, the location of the counter cation in the case of (**5**) leaves a small accessible void in the structure, that is, 9.8%, when compared to almost null with only 2.5% in (**5**).

### 3.2. Supramolecular Synthons as Structural Keys for the Comparison of The Crystal Structures

The supramolecular synthon concept has been employed for the description of the auto-assembly of the cluster units in the former crystal structures. The involved synthons in the five structures are reported in Table 2. One must mention that all involved synthons have been described in order to start from 0D Re cluster unit to reach 3D supramolecular frameworks. However, no hierarchy in the various synthons is required, and the chosen order of the synthons was governed by some logical rules for the simplest structural descriptions. The five crystal structures deserve then to be compared in order to highlight possible parameters that govern the building of the supramolecular architectures. 

First of all, it appears that in compounds (**1**), (**2**), (**4**), and (**5**), H_3_O_2_^−^ bridges, H bonds, and stacking govern the supramolecular frameworks. In contrast, in (**3**), which is the nitrate-based material, no H_3_O_2_^−^ bridge is involved, but NO_3_^−^ bridges participate in the supramolecular framework building. This may be due to the pH value of the starting solution containing nitrate gallium used for the synthesis of (**3**), which is around 2, whereas the pH value of the other four solutions is basic (9–11). This is in agreement with the pH dependence of the chemical stability of rhenium clusters for which a pH decrease favors the formation of aquahydroxo and hexaaqua cluster complexes [{Re_6_Q^i^_8_}(H_2_O)^a^_n_(OH)^a^_6-n_]^n–4^, whereas the hexahydroxo complex [{Re_6_Q^i^_8_}(OH)_6_^a^]^4−^ is stable at high pH values [43].

Secondly, the protonation of the cluster, and then the cluster unit charge, has a strong influence on the self-assembly of the cluster units. Indeed, for the negatively charged cluster unit [{Re_6_S^i^_8_}Pz^a^_2_(OH)^a^_3_(H_2_O)^a^]^−^ in (**4**) and (**5**) compounds, the supramolecular framework is built from two kinds of stackings that predominate compared to the other synthons (H_3_O_2_)^−^ bridge and H bonds. This is partly explained by the presence of crystallization water molecules that reinforce the framework. On the contrary, for the positively charged cluster unit [{Re_6_S^i^_8_}Pz^a^_2_(OH)^a^(H_2_O)^a^_3_]^+^ in (**3**), the main synthons are based on two kinds of H bonds. This behavior is well understood taking into consideration that this positively charged cluster unit contains three water molecules that promote H bonds as well as H_3_O_2_^−^ or NO_3_^−^ bridges. Moreover, the negatively charged unit is poorer in water molecules with only one molecule per cluster unit.

## 4. Conclusions

In conclusion, we have reported on the synthesis and crystal structure description of a series of hybrid clusters obtained from the substitution of OH^−^ groups in apical positions of the cluster unit [{Re_6_S^i^_8_}(OH)^a^_6_]^n−^ by pyrazine ligand units in the presence of various alkaline and alkaline earth metal salts. Changes in the overall formula charge of the resulting cluster units leading to [{Re_6_S^i^_8_}(Pz)^a^_2_(OH)^a^_4−x_(H_2_O)^a^_x_]^x−2^ allow changing the counter ion from cationic to anionic nature, while neutral units are also formed. The supramolecular frameworks have been described with the aim of the synthon concept and are built from H bonding and stacking interaction as well as in four complexes through H_3_O_2_^−^ bridges. It has been evidenced that the degree of protonation of the cluster unit, which depends on the pH of the solution, affects the self-assembling of the cluster units and consequently the resulting supramolecular interaction network. The self-assembling of the hybrid units is dominated by H-bonding interactions which predominate for the higher water contents. This study then clearly exemplifies the importance of the protonation and its control for the design of new hybrid cluster networks.

## Figures and Tables

**Table 1:** Crystal data and structure refinement parameters for **1**–**5**.

Empirical Formula	(1) C_8_H_8_N_4_O_6_Re_6_S_8_ (*trans*)	(2) C_8_H_8_N_4_O_4_Re_6_S_8_ (cis)	(3) C_12_H_12_N_7_O_10_Re_6_S_8_	(4) C_16_H_15_N_8_O_30.10_MgRe_12_S_16_	(5) C_8_H_8_N_4_O_12_KRe_6_S_8_
**Formula weight/g.mol^−1^**	1597.86	1597.86	1787.97	3572.71	1764.96
**Crystal description**	prism	prism	Stick	plate	prism
**Crystal color**	orange	orange	yellow	orange	orange
**Crystal size/mm^3^**	0.035 × 0.030 × 0.025	0.1 × 0.06 × 0.05	0.09 × 0.02 × 0.015	0.1 × 0.08 × 0.02	0.14 × 0.08 × 0.03
**Crystal system**	orthorhombic	triclinic	monoclinic	monoclinic	monoclinic
**Space group**	*F*2*dd* (No 43)	*P*$\bar{1}$(No 2)	*C*2/*c* (No 15)	*P*2_1_/*c* (No 14)	*C*2/*c* (No 15)
***a*/Å**	10.5588(8)	9.1971(4)	31.1128(15)	18.3212(5)	33.8720 (8)
***b*/Å**	15.3154(12)	18.7101(7)	16.0496(9)	12.6878(3)	12.6850(3)
***c*/Å**	40.2137(31)	19.9656(9)	16.5582(7)	30.1328(4)	18.2242(5)
***α/*°**	90.00	69.606(2)	90.00	90.00	90.00
***β/*°**	90.00	84.114(2)	115.788(2)	96.7635(12)	114.746(2)
***γ*/°**	90.00	88.969(2)	90.00	90.00	90.00
**Volume/Å^3^**	6503.0(9)	3202.7(2)	7444.9(6)	7110.6(3)	7111.3(3)
**Z**	8	4	8	4	8
**T/K**	150	150	150	150	150
***ρ*(calcd.)/g·cm^–3^**	3.264	3.314	3.190	3.337	3.297
***μ*/mm^–1^**	22.778	23.125	19.929	20.882	20.978
***F*(000)**	5552	2776	6328	6303	6216
***θ* range/°**	3.344 to 39.867	2.893 to 32.532	2.48 to 21.47	2.889 to 27.484	2.45 to 27.48
**Collected reflections**	21,131	42,407	25,810	104,690	27,818
**Independent reflections**	8219	22,614	8508	16,121	8152
**Observed reflections [*I* > 2σ(*I*)]**	4425	8786	3331	11,385	6229
**No. restraints/No. refined parameters**	4/138	0/398	0/353	0/762	0/353
**Goodness-of-fit on F^2^**	0.946	0.899	0.987	1.066	1.047
**R_1_, wR_2_**	0.0491, 0.1009	0.0536, 0.1148	0.0642, 0.1251	0.0423, 0.0850	0.0393, 0.1073
**R_1_, wR_2_ (all data)**	0.1206, 0.1228	0.1567, 0.1494	0.2056, 0.1647	0.0741, 0.0988	0.0574, 0.1193
**Larg. diff. peak and hole/e·Å^–3^**	2.645, −1.880	2.496, −2.462	2.227, −1.789	2.618, −2.061	3.265, −1.894

**Table 2:** Synthons and cluster units based on the {Re_6_S^i^_8_} core involved in the supramolecular networks of compounds (**1**)–(**5**).

Compound	Cluster Unit [{Re_6_S^i^_8_}(Pz)^a^_2_(OH)^a^_4−x_(H_2_O)^a^_x_]^x−2^	H_3_O_2_^−^ Bridge	H Bond	NO_3_^−^ Bridge	π-π Stacking
(**1**)	*trans-*{Re_6_S^i^_8_}(Pz)^a^_2_(OH)^a^_2_(H_2_O)^a^_2_	Strong H bond between cluster units → 2D framework	H bond between terminal nitrogen atoms of pirazine of cluster unit and equatorial oxygen atom from another cluster unit → 1D connectivity		
(**2**)	*cis-*{Re_6_S^i^_8_}(Pz)^a^_2_(OH)^a^_2_(H_2_O)^a^_2_	Strong H bond between cluster units along *a* axis → chains//*a*	H bond between terminal nitrogen atoms of pirazine of cluster unit and oxygen atom from another cluster unit → clusters tetramers, 2D framework		
		Strong H bond between cluster units perpendicular to *a* axis → 2D framework//(*bc*)			
(**3**)	[*cis-*{Re_6_S^i^_8_}(Pz)^a^_2_(OH)^a^(H_2_O)^a^_3_]		Strong H bonds between one free pyrazine group and two cluster units → 2D connectivity//(*ab*) of the chains, dimers	H bonds between one free nitrate group to two cluster units → chains//*b*	π-π stacking between pyrazine groups → cluster dimers
			H bonds between crystallization molecules themselves and also with O atoms from cluster units and nitrate groups		
(**4**)	[*cis*-Re_6_S^i^_8_(Pz)^a^_2_(OH)^a^_3_(H_2_O)^a^]	Strong H bond between O atoms of adjacent cluster units → 1D connectivity//*c* axis, cluster dimers	H bonds involving crystallization water molecules and water molecules connected to Mg^2+^ cation		π–π stacking between both aromatic rings of one cluster unit to connect with two different adjacent cluster units → chains//*a*
					π–π stacking between both aromatic rings of one cluster unit with only one another cluster unit belonging to another chains → cluster dimers, stacking of the chains along *b* axis
(**5**)	[*cis-*Re_6_S^i^_8_(Pz)^a^_2_(OH)^a^_3_(H_2_O)^a^]	Strong H bond between O atoms of adjacent cluster units → 1D connectivity//*a* axis, cluster dimers	H bonds involving crystallization water molecules and water molecules connected to K^+^ cation		π–π stacking between both aromatic rings of one cluster unit to connect with two different adjacent cluster units → chains//*c*
					π–π stacking between both aromatic rings of one cluster unit with only one another cluster unit belonging to another chains → cluster dimers, stacking of the chains along *b* axis

## Data Availability

Data are contained within the article and Supplementary Materials.

## Supplementary Materials

Additional representations of the crystal structures of (**3**) and (**4**) are available online in Figures S1–S6.
