# Supplementary figures and images for: Supramolecular Frameworks Based on Rhenium Clusters Using the Synthons Approach
Source: Molecules. 2021 May 1;26(9):2662. doi: 10.3390/molecules26092662 (PMC8125787; doi:10.3390/molecules26092662)

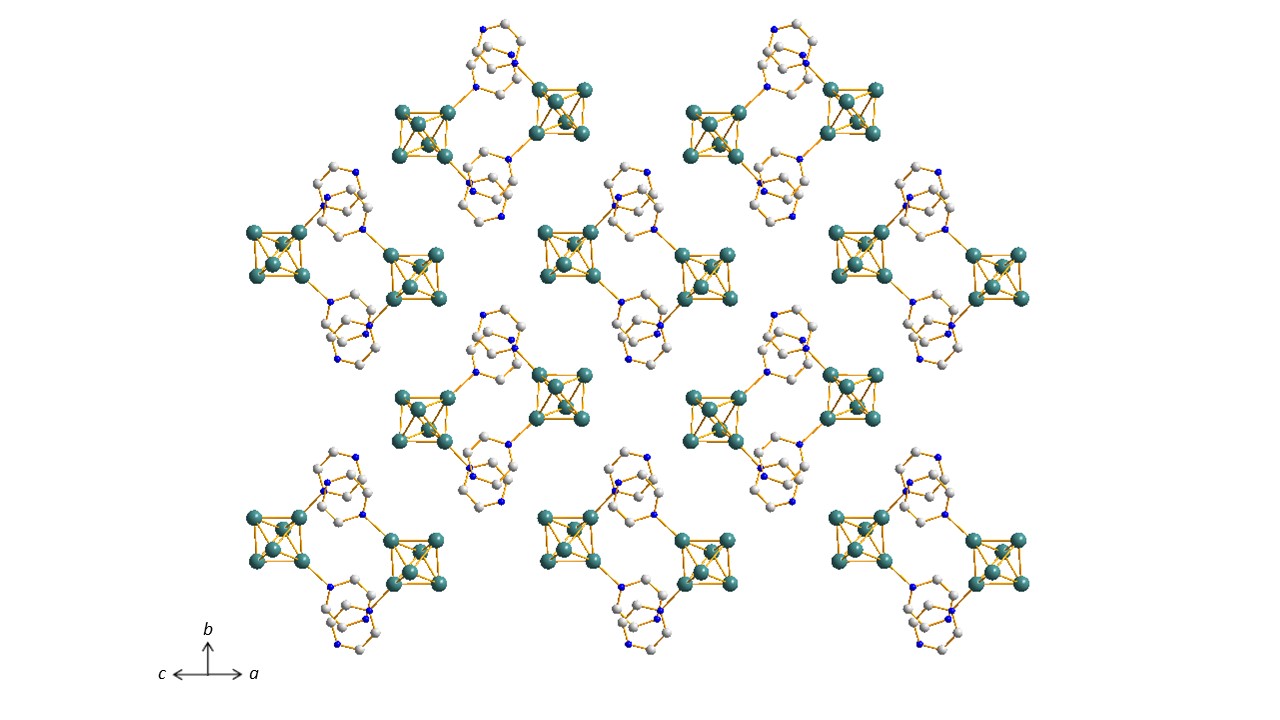

Supplement: Supplementary file 1 [file molecules-26-02662-s001.zip › FigS1.jpg]

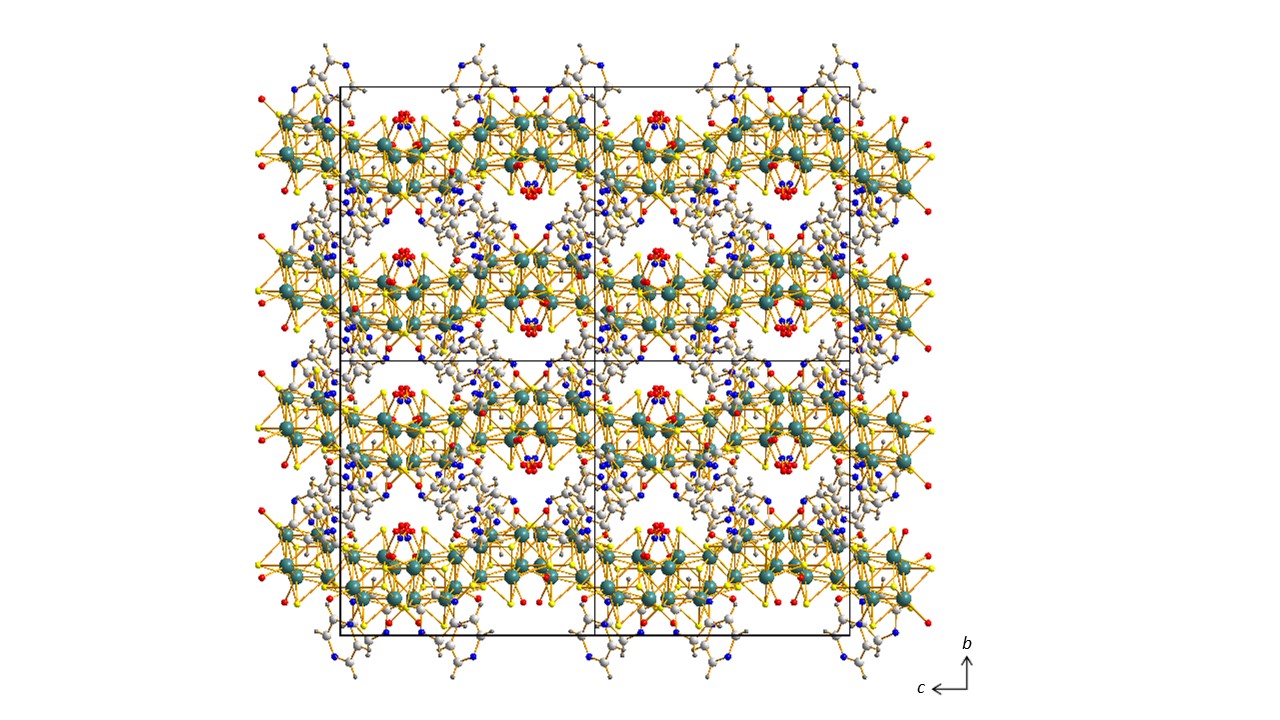

Supplement: Supplementary file 1 [file molecules-26-02662-s001.zip › FigS2.jpg]

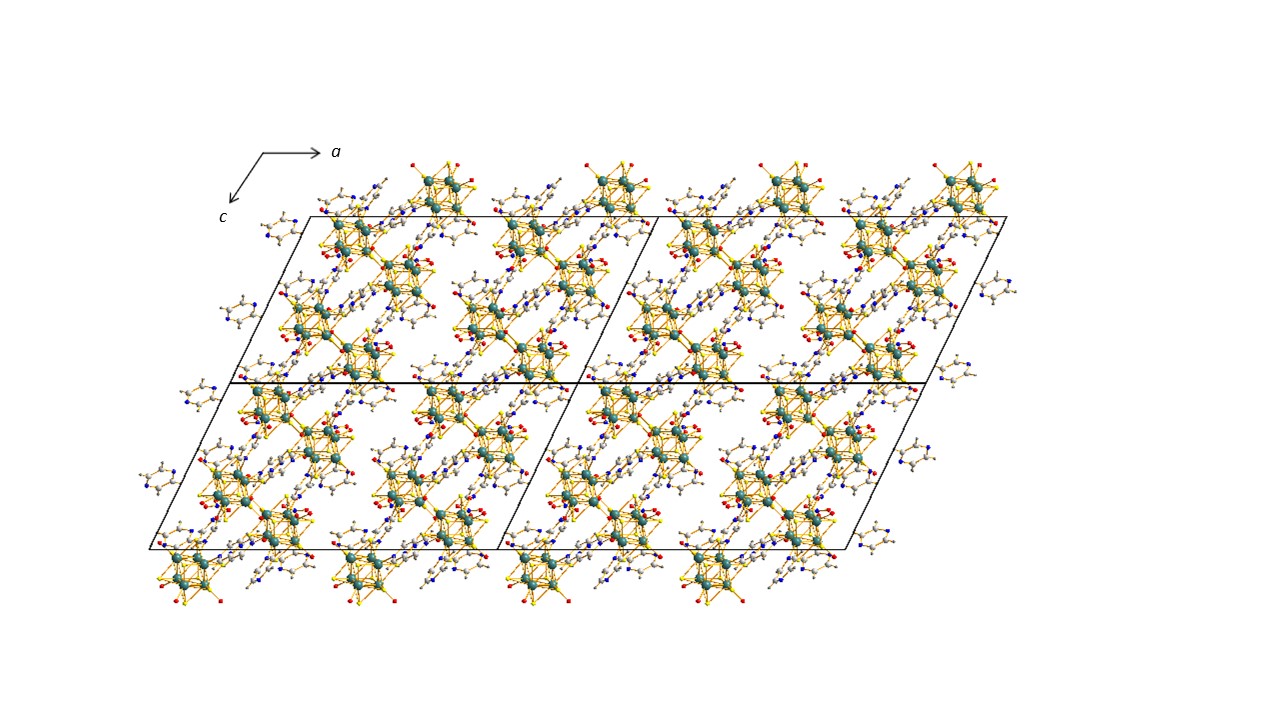

Supplement: Supplementary file 1 [file molecules-26-02662-s001.zip › FigS3.jpg]

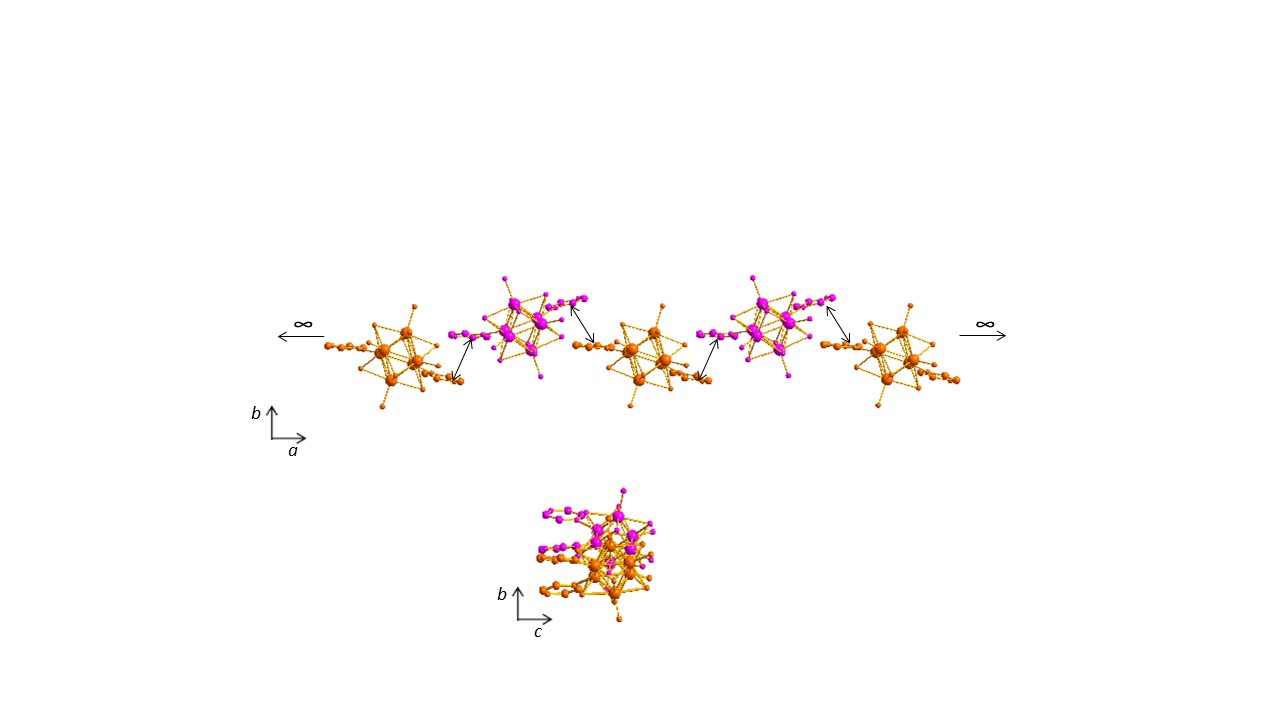

Supplement: Supplementary file 1 [file molecules-26-02662-s001.zip › FigS4.jpg]

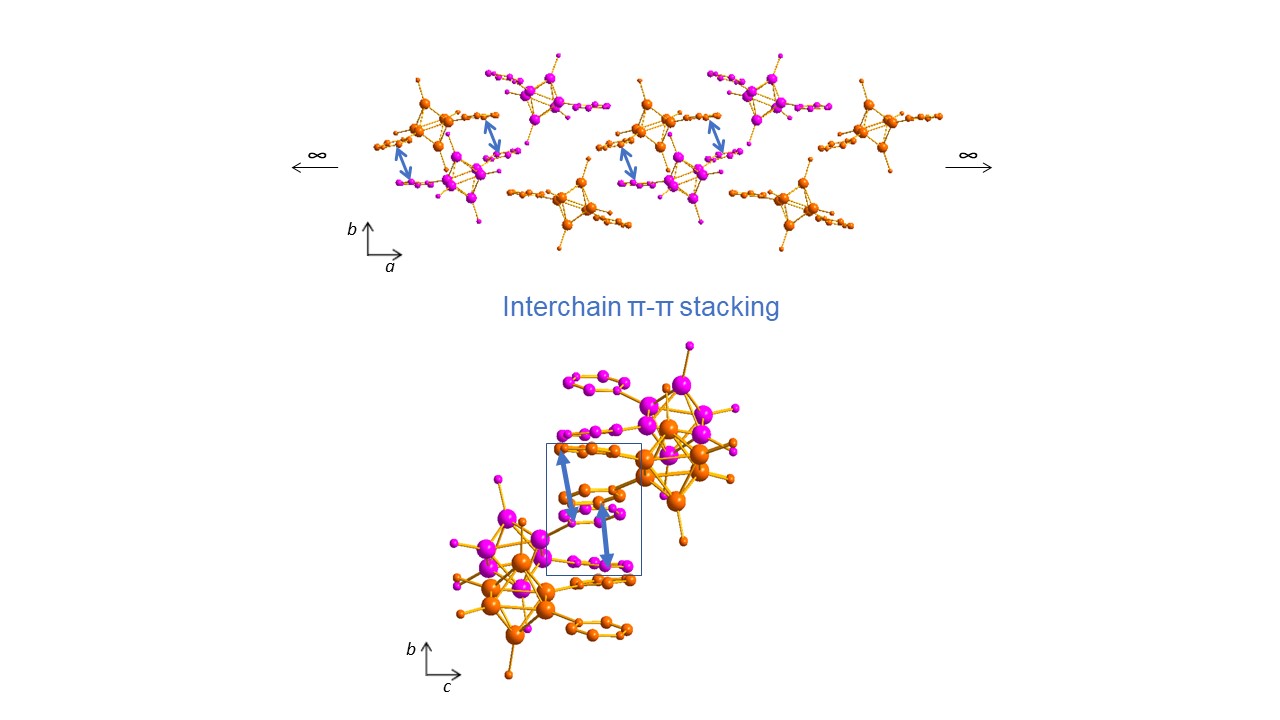

Supplement: Supplementary file 1 [file molecules-26-02662-s001.zip › FigS5.jpg]

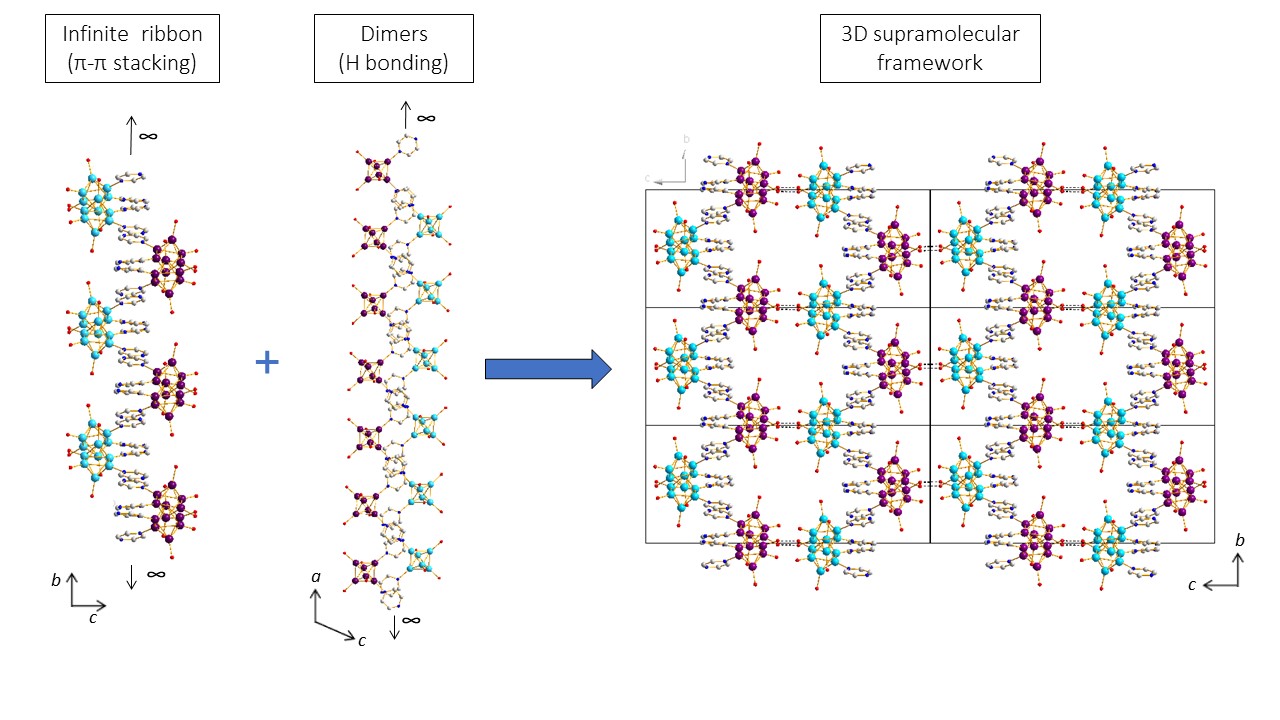

Supplement: Supplementary file 1 [file molecules-26-02662-s001.zip › FigS6.jpg]
